# P-1693. Comparison of Initial Treatment Strategies for Outpatients with Suspected Community Acquired-Pneumonia (CAP)

**DOI:** 10.1093/ofid/ofae631.1859

**Published:** 2025-01-29

**Authors:** Sheheryar Munir, Soonha Iqra, Suganthini Krishnan Natesan, Ryan Kuhn, Sorabh Dhar

**Affiliations:** Detroit Medical Center, DETROIT, Michigan; Jinnah Hospital karachi, DETROIT, Michigan; John D Dingell VAMC/Wayne State University, Detroit, Michigan; John D Dingell VA Medical Center, Detroit, Michigan; Wayne State University/Detroit Medical Center, John Dingell VAMC, Detroit, Michigan

## Abstract

**Background:**

Initial therapies for outpatient community acquired pneumonia (CAP) remain variable based on geographic location and resistance patterns. Infectious Diseases Society of America (IDSA) guidelines recommend either monotherapy for patients who lack comorbidities or risk factors for resistant organisms, or combination therapy for those at risk for severe pneumonia. Despite clinical guidance, it remains challenging for providers to prescribe guideline recommended therapies. Assessments of local practices and outcomes are essential for optimal CAP management.

Table 1

**Figure:**
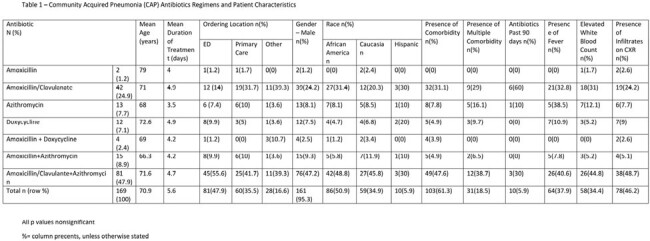

Community Acquired Pneumonia (CAP) Antibiotics Regimens and Patient Characteristics

**Methods:**

A retrospective review of outpatient antibiotics prescribed from 1/2023 to 3/2024 for the indication of pneumonia was conducted at a Veterans Affairs Hospital. Clinical and antibiotic data were collected and analyzed to for comorbidities, risk factors, and outcomes, and assessed for indications for monotherapy or combination therapies in concordance with current IDSA guidelines. Antibiotics assessed included amoxicillin, doxycycline, azithromycin, amoxicillin/clavulanate either as monotherapy or combination therapy (beta-lactam antibiotic + azithromycin or doxycycline). Outcomes were compared between the 2 groups.

Table 2

**Figure:**
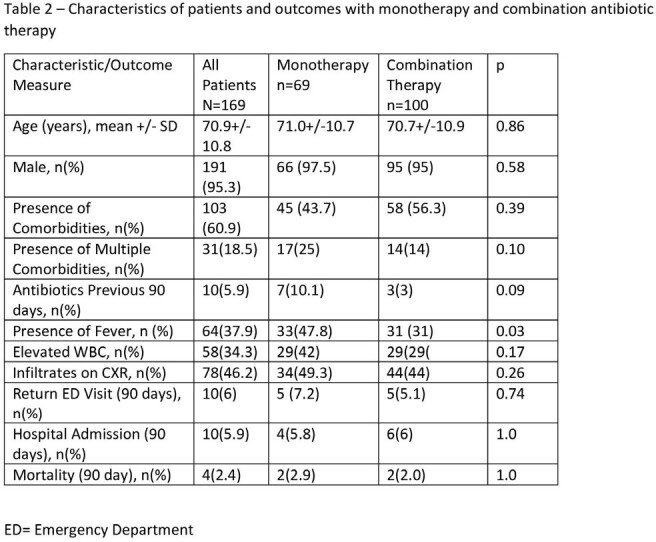

**Results:**

A total of 169 patients were reviewed of which 103 (60%) had comorbidities or risk factors for MRSA and pseudomonas. Most common antibiotics prescribed were amoxicillin/clavulanate + azithromycin in 81 (47.9%) and amoxicillin/clavulanate in 42 (24.9%) of patient (table 1). Monotherapy was prescribed in 40.8% of patients of whom 24 (34.8%) had no comorbidities or risk factors for resistance, while combination therapy prescribed in 100 (59.2%) patients of whom 58 (56.3%) possessed risk factors for resistance (p=0.4). There were no other differences noted in the clinical characteristics, risk factors, or outcomes between the monotherapy and combination therapy group.

**Conclusion:**

Antibiotic prescribing for CAP was found to be variable with low concordance with guideline recommendations for monotherapy and combination therapies. Despite variability in regimens, no differences were noted in risk factors or outcomes suggesting that regional practices dictate CAP antibiotic selection. Appropriate prescribing patterns need to be assessed by stewardship programs.

**Disclosures:**

**All Authors**: No reported disclosures

